# Clomazone impact on fungal network complexity and stability

**DOI:** 10.3389/fmicb.2023.1124127

**Published:** 2023-01-26

**Authors:** Hairong He, Jiarui Huang, Zhenzhu Zhao, Weisheng Feng, Xiaoke Zheng, Pengqiang Du

**Affiliations:** ^1^College of Pharmacy, Henan University of Chinese Medicine, Zhengzhou, China; ^2^College of Plant Protection, Henan Agricultural University, Zhengzhou, China

**Keywords:** clomazone, fungi, network, dissimilarity, stability

## Abstract

**Introduction:**

Soil fungal network composition and stability are important for soil functions, but there is less understanding of the impact of clomazone on network complexity and stability.

**Methods:**

In this work, two agricultural soils were used to investigate the impact of clomazone on fungal network complexity, composition, and stability. The two soils were treated with clomazone solution (0, 0.8, 8, and 80  mg kg^−1^) and kept in an incubator.

**Results and Discussion:**

Under the influence of clomazone, the fungal network nodes were decreased by 12–42; however, the average degree was increased by 0.169–1.468 and fungal network density was increased by 0.003–0.054. The keystone nodes were significantly changed after clomazone treatment. Network composition was also impacted. Specifically, compared with control and clomazone treatments in both soils, the shared edges were fewer than 54 in all comparisons, and network dissimilarity was 0.97–0.98. These results suggested that fungal network composition was significantly impacted. The network robustness was increased by 0.0018–0.0209, and vulnerability was decreased by 0.00018–0.00059 in both soils, which indicated that fungal network stability was increased by clomazone. In addition, the functions of network communities were also changed in both soils. These results indicated that clomazone could significantly impact soil fungal networks.

## 1. Introduction

Soil fungi are important for earth element cycling, and they are important participants, decomposers, mediators, and undertakers in ecosystems (Tedersoo et al., 2014). Complicated microorganism relationships occur in soil fungi, such as mutualism, commensalism, parasitism, neutralism predation, competition, and amenalism (Faust and Raes, 2012; Coyte et al., 2015). Through these diverse relationships, an organic entity is formed. Therefore, interactions between microbes are vital for maintaining homeostasis in soil processes.

In modern agricultural practice, pesticides are necessary and a widespread interference factor in soil fungal community structures (Du et al., 2021). Previous work reported that pesticides could impact soil microbial abundance, microbial community, and functions (Lerner et al., 2020; Li et al., 2020; Liu et al., 2020; Qiao et al., 2020; Yang et al., 2021). Microbial connections are important for soil function preservation. Altered microbial communities will change the connections of species (Du et al., 2021). Network analysis has been increasingly used in microbial ecology to evaluate these complicated relationships (Berry and Widder, 2014; Przulj and Malod-Dognin, 2016). In the process of environmental change, analysis of the impact of microbial networks could evaluate the stability of their composition and functions. For example, Wu M. H. et al. (2021) reported that permafrost degradation reduced microbial network stability and increased carbon loss, and Shen et al. (2022) analyzed the impact of plant diversity on soil fungal network stability and functions. Previous researchers have focused on the impact of pesticide on topological indexes (Gao et al., 2018; Xun et al., 2021; Su et al., 2022), but this limited researchers from exploring the impact on microbial networks. Slight changes to topological indexes may due to the same number of changed individuals. Analysis of persistent species and changes in network composition in response to stresses has important implications for soil community functions. For example, the genes related to nitrogen and phosphorus metabolism are the main genes for soil microbial community stability (Xun et al., 2021). In addition, network stability is important in evaluating the resistance of microbial networks to interference. However, no research has been carried out concerning microbial network node persistence, composition, and stability in pesticide-polluted soils.

Clomazone is an isoxazolidinone compound commonly used as a selective herbicide for many crops, and it has a half-life of >195 days in the field (PPDB, 2023). Previous studies have reported that clomazone could negatively impact soil fungal communities, indicating that network structures can be damaged (Du et al., 2018). However, no research about fungal networks has been conducted. Therefore, to evaluate the influence of clomazone on fungal networks, we carried out a microcosmic experiment indoors over a period of 3 months. For this purpose, fungal network complexity, stability, dissimilarity, and the related functions were evaluated. Network complexity was evaluated by the number of nodes and links, the average degree of nodes, network density, and clustering coefficient. Mo et al. (2021) studied the impact of salinity shifts on microeukaryotic plankton communities through networks. Yuan et al. (2021) researched the influence of warming on bacterial network complexity, stability, preserved modules, network nodes, and community functions. Network stability is important for resistance to stresses and performing functions, and it is evaluated *via* robustness and vulnerability in this study (Deng et al., 2012; Yuan et al., 2021). de Vries et al. (2018) evaluated soil fungal and bacterial network stability, and found fungal networks were more stable than bacterial networks. Fungal network dissimilarity was also evaluated using the shared nodes and edges between two microbial networks (Poisot et al., 2012; Mo et al., 2021). Our aims were to clarify whether clomazone could influence fungal network composition and stability.

## 2. Materials and methods

### 2.1. Experimental design

Two soil textures were used for experimentation. Samples of silty clay soil (classified based on soil particle diameters) were obtained from the Jiansanjiang reclamation area, Heilongjiang province (JSJ), and samples of silty loam soil were obtained from Langfang research base, Hebei province (LF). For JSJ soil, organic matter was 18.0 g kg^−1^, available P was 74.9 mg kg^−1^, available K was 289.8 mg kg^−1^, and pH was 7.07; for LF soil, organic matter was 25.8 g kg^−1^, available P was 51.7 mg kg^−1^, available K was 28 mg kg^−1^, and pH was 7.24. The soils were sieved through a 2-mm mesh and preincubated for 2 weeks (Trabue et al., 2006). Three concentrations of clomazone were used to treat the soils: 0.8 mg kg^−1^ [active ingredients (a.i.) per soil dry weight (dw)], 8 mg kg^−1^, and 80 mg kg^−1^, here referred as L, M, and H and corresponding to 1, 10, and 100 times the recommended application rate, respectively. The M level represents excessive use in the field, and the H level represents extreme contamination in soil (e.g., soil near a pesticide factory). These concentrations have also been carried out in other studies (Crouzet et al., 2010; Muñoz-Leoz et al., 2011; Wu C. et al., 2021). The concentration was based on a soil depth of 10 cm with a bulk density of 1.5 g cm^−3^ (Cheng et al., 2014). The purity of clomazone was 98% having been dissolved in acetone (analytical grade, Beijing Chemical Company). 100 μL of clomazone solution was added to bottles with 50 g of soil and thoroughly mixed for 15 min. After that, 200 g of soil was added to each bottle and mixed for 15 min at 3,000 rpm. For the control group, soil samples were treated with carrier solution lacking clomazone. Each treatment was carried out in triplicate. Soil moisture was adjusted continuously by adding deionized water to 50% of the water holding capacity during the whole period. A weighing method was used to determine the loss of water every 2 days, and the soil moisture was kept according to the lost weight. The brown bottles containing the treated soils were stored in an artificial climate box for 90 days at 25°C. Soil samples (about 15 g) were taken at 7, 15, 30, 60, and 90 days, and stored at −80°C until analysis.

### 2.2. Characterization of soil fungal communities

A PowerSoil Isolation kit (Mo Bio Laboratories, Carlsbad, CA, United States) was used on 0.5 g of soil to extract microbial DNA. Microbial DNA quality was evaluated with a ND-1000 spectrophotometer (NanoDrop Technologies) based on the ratios of absorbance measured as follows: 260/230 nm and 260/280 nm. The forward primer was ITS3_KYO2 (5′-GATGAAGAACGYAGYRAA-3′) and the reverse primer was ITS4 (5′-TCCTCCGCTTATTGATATGC-3′; Tian et al., 2017). Microbial DNA was amplified *via* PCR reactions, and each PCR solution contained 100–300 ng of DNA template, 1.5 μL of each 10 μM primer, 1 μL of KOD-Plus-Neo enzyme (Toyobo, Shanghai, China), 5 μL of 2 mM dNTPs, 5 μL of 10× PCR Buffer for KOD-Plus-Neo, 3 μL of 25 mM MgSO_4_, and water to make 50 μL. The PCR cycling program was as follows: an initial step at 94°C for 2 min, followed by 35 cycles of 98°C for 10 s, 62°C for 30 s, and 68°C for 30 s, with a final extension at 68°C for 10 min. A negative control without DNA templates was also settled. The PCR products were purified with a PCR Purification kit from Qiagen (Hilden, Germany) after analysis using 1.5% agarose gel electrophoresis. The concentration of PCR products was determined using a Qubit 3.0 fluorometer (Life Technologies, Waltham, MA, United States). Purified PCR products were sequenced with the Illumina platform (Santiago, CA, United States) and a 2 × 250 bp kit. Amplicon sequencing data were processed with USEARCH (Edgar, 2010, 2013), and clean data were clustered into operational taxonomic units (OTUs) with similarity set at 97%.

### 2.3. Network construction and characterization

All co-occurrence networks were constructed on the basis of Pearson correlations of fungal OTUs abundance, and performed on Cytoscape software by CoNet plugin (Faust and Raes, 2016). A Pearson’s correlation coefficient (*r*) of ≥0.7 or ≤−0.7 was used to evaluate the associations of pairwise fungal OTUs. The following network topological indexes were calculated with the Gephi platform: total nodes, total links, average degrees, network density, modularity, clustering coefficient, and path lengths. Network nodes were the OTUs in the network. Network density was the ratio of actual edges and capable edges in the network. Degree represents the connections of a node to other nodes, and modularity is based on the connections between nodes and represents the degree to which a network is divided into different modules (Strogatz, 2001). The networks were established using the Gephi platform.

The topological role of each node was based on its within-module connectivity (Zi) and among-module connectivity (Pi; Guimerà and Nunes Amaral, 2005). According to previous studies (Olesen et al., 2007; Chen et al., 2019), the nodes were classified as module hubs (Zi ≥ 2.5, Pi < 0.62), connectors (Zi < 2.5, Pi ≥ 0.62), and network hubs (Zi ≥ 2.5, Pi ≥ 0.62), and they were referred to as keystone nodes (Banerjee et al., 2019; Röttjers and Faust, 2019).

### 2.4. Network stability

Normally, network stability is evaluated *via* robustness and vulnerability (Wu M. H. et al., 2021; Yuan et al., 2021). Robustness is defined as the remaining proportion of species of the network after random removal of some nodes (Montesinos-Navarro et al., 2017). In this study, each 0.05% of nodes randomly removed simulated random species removal. Vulnerability is also an index used to evaluate network stability based on node removal (Yuan et al., 2021).

### 2.5. Network composition dissimilarity

In this study, we used several indexes to evaluate the dissimilarity of fungal networks between different treatments. Shared nodes and edges between two networks were used to evaluate coexisting elements of different networks. Network dissimilarity based on network nodes and edges has been used to evaluate network differences (Poisot et al., 2012; Mo et al., 2021).

### 2.6. Fungal functions

FUNGuild was used to predict the functions of fungi from the amplicon sequencing data (Nguyen et al., 2016). According to trophic modes, there were three categories for fungi, namely, pathotroph, saprotroph, and symbiotroph. In order to take advantage of fungal functions, these three categories were further divided. Pathotroph was divided into animal pathogen, plant pathogen, fungal parasite, lichen parasite, bryophyte parasite, and endophyte; saprotroph was divided into dung saprotroph, leaf saprotroph, plant saprotroph, soil saprotroph, and wood saprotroph; symbiotroph was divided into ectomycorrhizal, ericoid mycorrhizal, and endophyte. The relative abundance of sub-groups of the fungal functions was used to evaluate the networked fungal OTUs community. The Mantel test was used to evaluate the relationships of fungal communities to fungal functions (Duan et al., 2020).

## 3. Results

### 3.1. Network indexes and composition

**Figure 1:**
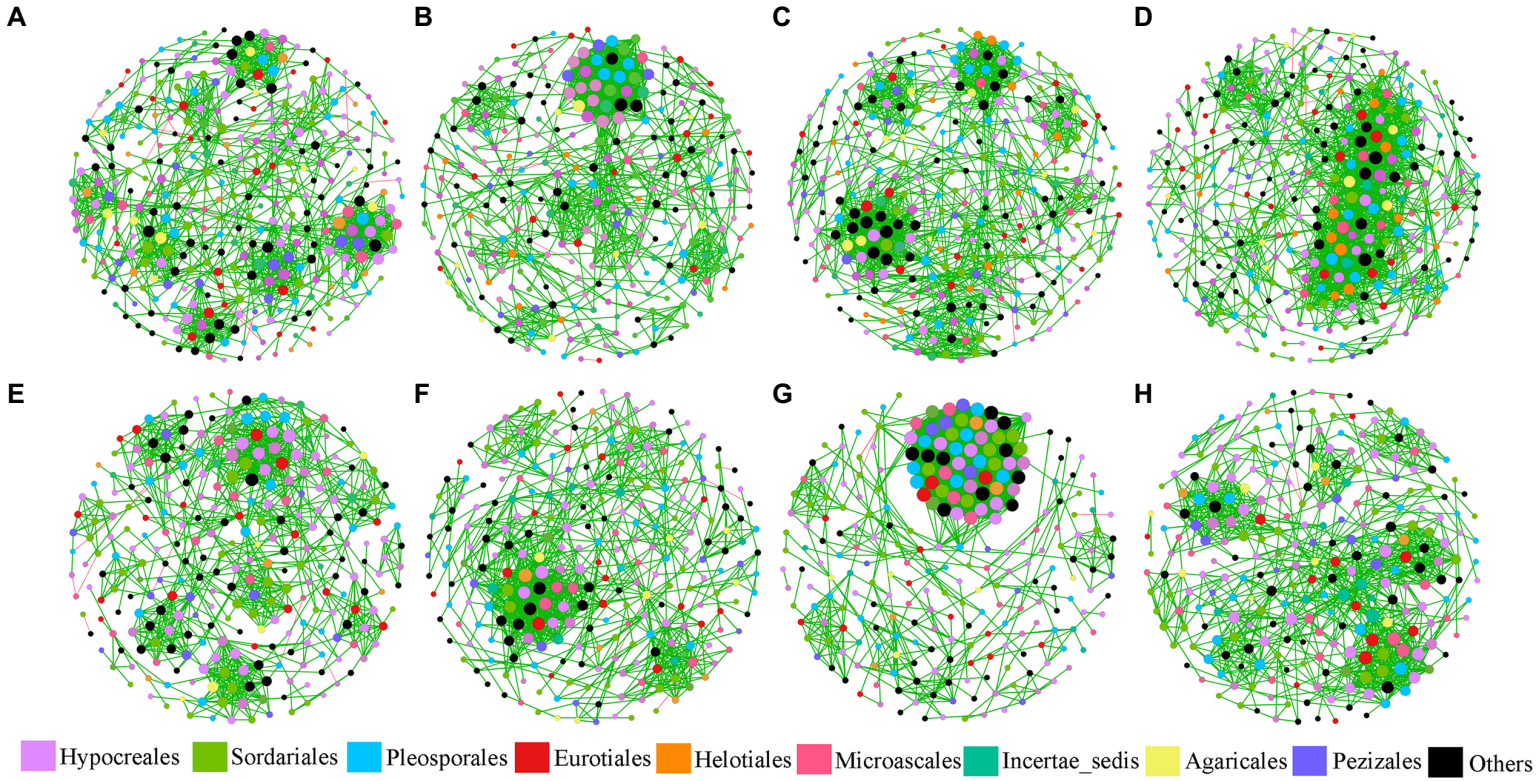
Visualization of fungal networks for each treatment in two soils. **(A–D)** are control, L, M, and H treatments in JSJ soil, respectively; and **(E–H)** are control, L, M, and H treatments in LF soil, respectively.

There were 2,387 fungal OTUs that were identified and used in this study. Eight fungal networks were established for each treatment based on Pearson’s correlation coefficients of fungal OTUs (Figure 1), and Table 1 lists each network’s topological indexes. Compared with the control group, the total nodes decreased by 42, 12, and 15 in L, M, and H treatment in JSJ soils, and 14, 27, and 17 in L, M, and H treatment in LF soils, respectively. These network nodes were assigned to nine dominant fungal orders in both soils, which were Hypocreales, Sordariales, Pleosporales, Eurotiales, Microascales, Pelotiales, Helotiales, Incertae sedis, and Agaricales. However, the average degree was increased by 0.834, 1.251, and 3.202 in L, M, and H treatment in JSJ soils, and 0.319, 9.553, and 1.041 in L, M, and H treatment in LF soils, respectively. Network density was increased by 0.005, 0.005, and 0.012 in L, M, and H treatment in JSJ soils, and by 0.003, 0.054, and 0.007 in L, M, and H treatment in LF soils, respectively. In addition, the percentage of positive edges increased by 0.54%–0.99% in all clomazone-treated JSJ soil, and increased by 0.19%–0.69% in all clomazone-treated LF soil.

**Table 1:** Topological indexes for each network in JSJ and LF soils.

	JSJ				LF			
	CK	L	M	H	CK	L	M	H
Total nodes	346	304	334	331	279	265	252	262
Total links	1,267	1,240	1,432	1742	1,040	1,030	2,143	1,113
Average degree	7.324	8.158	8.575	10.526	7.455	7.774	17.008	8.496
Modularity	0.807	0.769	0.814	0.835	0.81	0.777	0.781	0.775
Path length	5.813	5.776	5.291	4.585	5.348	5.374	6.138	4.771
Clustering coefficient	0.447	0.485	0.479	0.458	0.459	0.444	0.561	0.469
Network density	0.023	0.028	0.028	0.035	0.029	0.032	0.083	0.036
Number of positive edges	1,250	1,234	1,427	1728	1,031	1,023	2,138	1,111
Percentage of positive edges (%)	98.66%	99.52%	99.65%	99.20%	99.13%	99.32%	99.77%	99.82%
Number of negative edges	17	6	5	14	9	7	5	2
Percentage of negative edges (%)	1.34%	0.48%	0.35%	0.80%	0.87%	0.68%	0.23%	0.18%

### 3.2. Network keystone nodes

**Figure 2:**
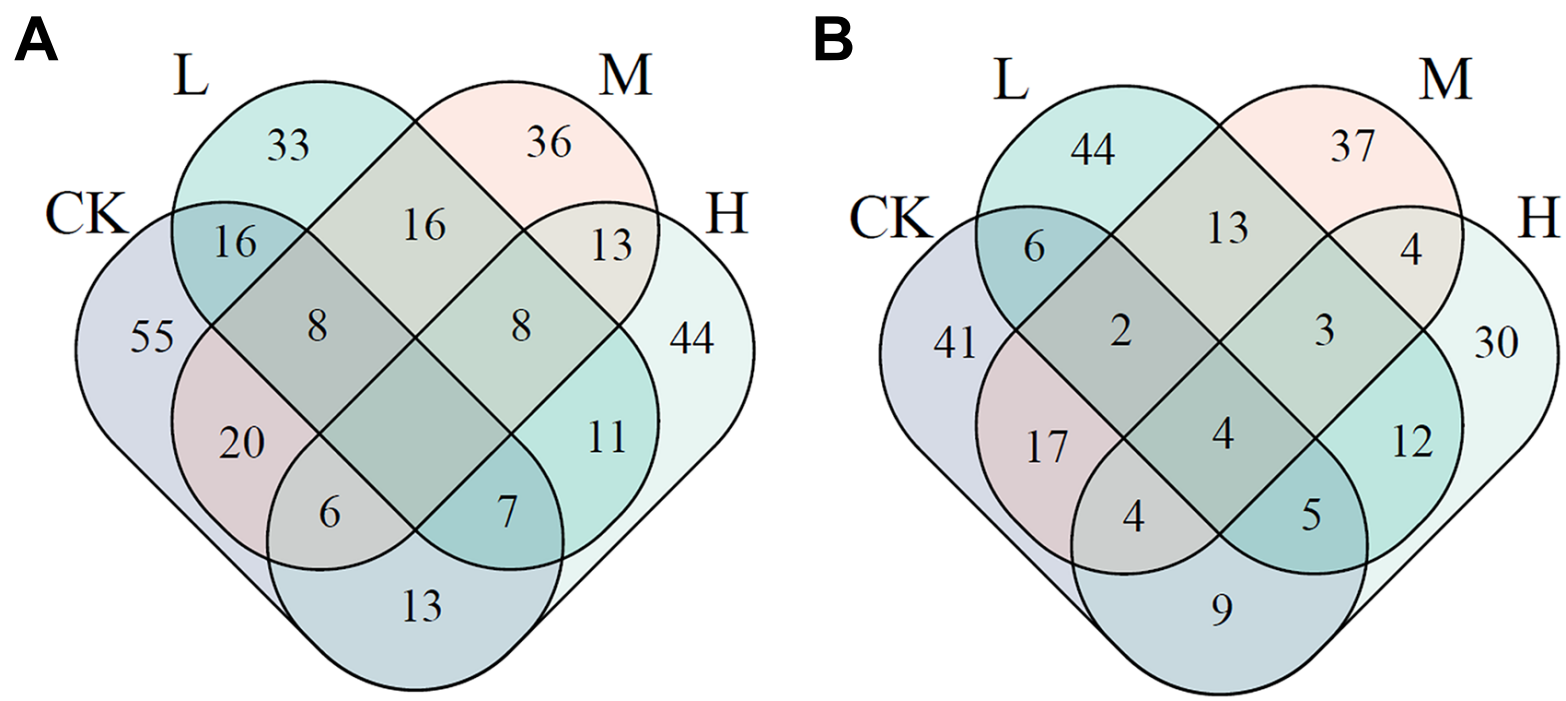
Venn diagram of keystone nodes in each network in JSJ **(A)** and LF **(B)** soils.

On the basis of nodes’ Zi and Pi, the number of keystone nodes decreased by 19–27 in JSJ soil treated with clomazone. In LF soil, it increased by 1 in L treatment, but decreased by 4 and 17 in M and H treatments. The shared keystone nodes between the control and clomazone treatments were 31, 34, and 26 in JSJ soil, and 17, 27, and 22 in LF soils, respectively (Figure 2).

### 3.3. Network dissimilarity

The network dissimilarity, shared nodes and edges are shown in Table 2. In JSJ soil, there were 260, 269, and 271 shared nodes in comparison to control-L, control-M, and control-H in JSJ soil, respectively. There were 210, 208, and 211 shared nodes in comparison to control-L, control-M, and control-H in LF soil, respectively. Compared with the nodes in each network, the shared nodes accounted for significant percentages of the nodes in each network. However, the shared edges between the control and clomazone-treated soil were significantly low. There were 23, 27, and 24 shared edges in comparison to control-L, control-M, and control-H in JSJ soil, respectively; and there were 17, 54, and 27 shared nodes in comparison to control-L, control-M, and control-H in LF soil, respectively. The dissimilarity between different networks in the two soils were both high, in the range of 0.97–0.98.

**Table 2:** Numbers of shared nodes and edges and their dissimilarity between control and each clomazone treatment in JSJ and LF soils.

	Shared nodes	Shared edges	Dissimilarity of networks
*JSJ*			
CK vs. L	260	23	0.98
CK vs. M	269	27	0.98
CK vs. H	271	24	0.98
*LF*			
CK vs. L	210	17	0.98
CK vs. M	208	54	0.97
CK vs. H	211	27	0.97

### 3.4. Network stability

**Figure 3:**
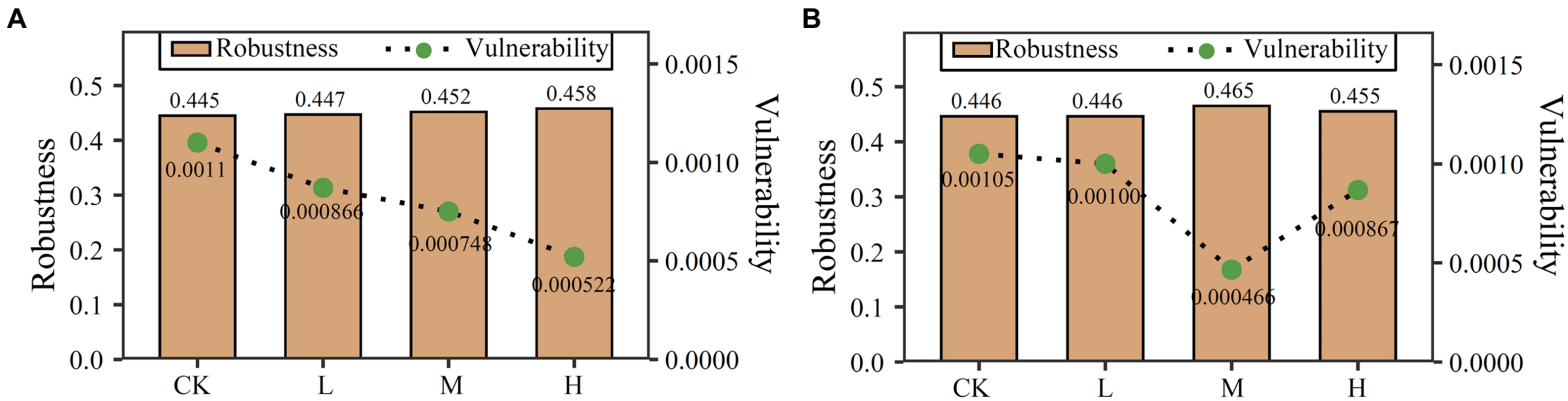
Robustness and vulnerability of each network in JSJ **(A)** and LF **(B)** soils.

On the basis of random species loss, network robustness was increased by clomazone in the two soils. In JSJ soil, it was increased by 0.0018, 0.0067, and 0.0125 in L, M, and H, respectively. In LF soil, it was increased by 0.0209 and 0.0103 in M and H, respectively (Figure 3). Vulnerability was decreased by clomazone in both soils. In JSJ soil, it was decreased by 0.00029, 0.00035, and 0.00057 in L, M, and H, respectively. In LF soil, it was decreased by 0.000047, 0.00059, and 0.00018 in L, M, and H, respectively (Figure 3).

### 3.5. Connection of fungal network patterns to functions

**Figure 4:**
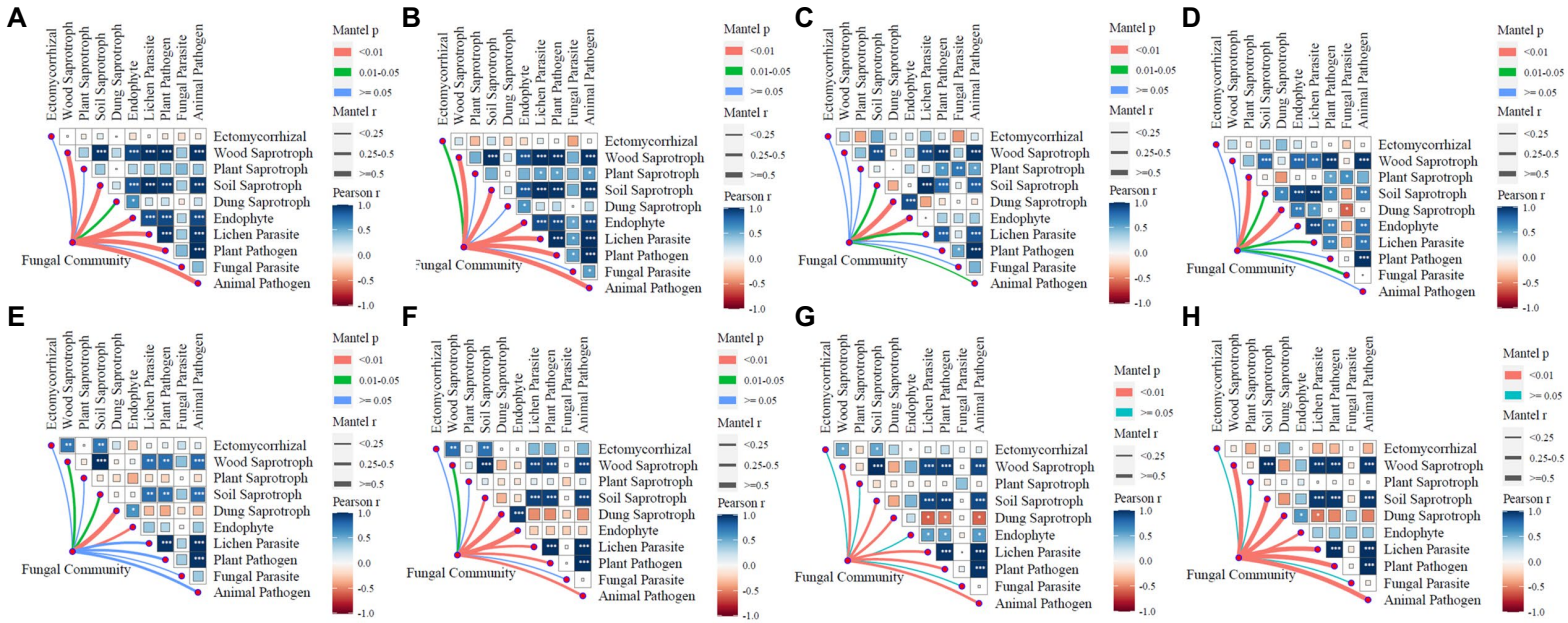
Relationships between fungal network communities and functions for each treatment in two soils. **(A–D)** are control, L, M, and H treatments in JSJ soil, respectively; and **(E–H)** are control, L, M, and H treatments in LF soil, respectively.

There were 10 guilds that were identified in this study: ectomycorrhizal, wood saprotroph, plant saprotroph, soil saprotroph, dung saprotroph, endophyte, lichen parasite, plant pathogen, fungal parasite, and animal pathogen. The correlation of network communities between these 10 functions are shown in Figure 4. In JSJ soil, the correlation profiles of fungal communities to functions were the same for control and L treatments, and were correlated to wood saprotroph, soil saprotroph, plant pathogen, animal pathogen, lichen parasite, and endophyte; M treatment was correlated to dung saprotroph and endophyte; H treatment was correlated to dung saprotroph. In LF soil, fungal communities were significantly correlated to dung saprotroph in the control treatment, and also significantly correlated to dung saprotroph and endophyte in L treatment; M and H treatments were significantly correlated to wood saprotroph, lichen parasite, plant pathogen, and animal pathogen.

## 4. Discussion

Soil environments are ecological systems. Soil fungi are important and play key roles as decomposers, mutualists, plant pathogens, C cycling mediators, and nutrient moderators (Tedersoo et al., 2014). Complicated relationships exist among fungi, including mutualism, competition, parasitism, and inhibition/amenalism. Microorganism networks have been used to analyze these complicated relationships and evaluate the influence on microorganism connections (Mo et al., 2021). These works have suggested that networks are an effective way to research the relationships of microorganisms (Ze et al., 2013; Przulj and Malod-Dognin, 2016).

In this work, influenced network complexities indicated that the relationships of fungal species were impacted. Specifically, the decreased total nodes in clomazone networks suggested that clomazone disconnected more species from others in fungal communities. This was mostly due to the fact that more connections among species were impacted by clomazone. In the study by Zhang et al., heavy Cu also decreased fungal interactions (Zhang et al., 2022). However, the increased average degree and network density suggested that network nodes were more connected with others in one network. It also suggested that species in the network more frequently communicated with others using clomazone. Mesosulfuron-methyl also increased microorganisms’ communication among species and induced an increase in average degrees and network density in different soils (Du et al., 2021). The different profiles of keystone nodes suggested that network nodes’ topological roles were also significantly changed by clomazone. In addition, the increased positive edges suggested that clomazone induced increased relationships of mutualism, commensalism, parasitism, and neutralism predation (Faust and Raes, 2012; Coyte et al., 2015).

Influenced network complexities indicated that soil fungal network composition was impacted. The results of network dissimilarity and shared nodes confirmed this inference. Network dissimilarity was first published by Poisot et al. (2012), and has also been used by other researchers (Mo et al., 2021; Liao et al., 2023). For example, Liao et al. (2023) analyzed the differences between marine medaka gut and gill microbial networks using network dissimilarity; in the study by Mo et al. (2021), shared nodes, edges, and network dissimilarity were used to evaluate the differences of microeukaryotic plankton networks in different salinities in subtropical urban reservoirs. These results suggested that network dissimilarity is a valuable method to evaluate the differences of two networks. The significantly high dissimilarity suggested that fungal network composition has been significantly changed by clomazone in the two soils.

Influenced network complexities and composition indicated that soil fungal network stability and functions were impacted. Soil fungal network stability is important in performing functions, maintaining ecosystem sustainability, and for environmental protection (Coyte et al., 2015; Pan et al., 2023). Microorganism network stability is always evaluated using robustness and vulnerability. In this work, the increased network robustness and decreased vulnerability indicated that fungal networks were more stable in the two clomazone-treated soils. These results suggested that fungal networks were more resistant to disturbances after clomazone treatment (McCann, 2000). This was obviously due to clomazone increasing edges in the networks (Yuan et al., 2021), and it also suggested that fewer species were lost from the more connected networks. In addition, this result also suggested that it is hard for fungal networks to return to their original state.

The impacts above were also shown on network-related functions. In JSJ soil, the network community was significantly related to 6, 2, and 1 function in control, M, and H treatments, respectively. These results suggested that large amounts of clomazone impacted fungal network community functions, and function diversity also decreased in JSJ soil. However, function diversities related to the network community were increased in LF soil by clomazone, mostly due to different fungal compositions. Soil fungal functions sensitive to herbicide has been proven in other studies (Flores et al., 2014; Chen et al., 2022). In the study by Chen et al. (2022), oxathiapiprolin significantly impacted soil fungal community functions in an indoor experiment. Clothianidin, imazalil, and diazinon also showed negative effects on stream fungi, and they also influenced organic matter processing and energy cycling (Flores et al., 2014; Huang et al., 2021).

## 5. Conclusion

In this study, the impact of clomazone on soil fungal networks was evaluated *via* network complexities, composition, keystone nodes, and stability. Clomazone decreased fungal network nodes, but increased the average degree and network density. Fungal network composition and keystone nodes were also impacted by clomazone. Increased robustness and decreased vulnerability suggested that network stability was increased by clomazone. Fungal network community functions were also impacted in both soils. Overall, clomazone could significantly influence fungal networks.

## Data availability statement

Publicly available datasets were analyzed in this study. This data can be found at: https://www.ncbi.nlm.nih.gov/biosample. BioSample: SAMN08721648-SAMN08721767.

## Author contributions

HH and PD conceived and wrote this work. HH, PD, and JH performed the bioinformatics analyses. ZZ, WF, XZ, and HH revised this work. All authors contributed to the article and approved the submitted version.

## Funding

This work was supported by the National Natural Science Foundation of China (30900443).

## Conflict of interest

The authors declare that the research was conducted in the absence of any commercial or financial relationships that could be construed as a potential conflict of interest.

## Publisher’s note

All claims expressed in this article are solely those of the authors and do not necessarily represent those of their affiliated organizations, or those of the publisher, the editors and the reviewers. Any product that may be evaluated in this article, or claim that may be made by its manufacturer, is not guaranteed or endorsed by the publisher.
